# CYP1A1 protein activity is associated with allelic variation in pterygium tissues and cells

**Published:** 2012-07-18

**Authors:** Mei-Ling Peng, Yi-Yu Tsai, Chun-Chi Chiang, Ying-Che Huang, Ming-Chih Chou, Kun-Tu Yeh, Huei Lee, Ya-Wen Cheng

**Affiliations:** 1 Institute of Medicine, Chung Shan Medical University, Taichung, Taiwan; 2 Department of Ophthalmology, Chung Shan Medical University Hospital, Taichung, Taiwan; 3 Department of Ophthalmology, China Medical University Hospital, Taichung, Taiwan; 4 School of Medicine, Chung Shan Medical University, Taichung, Taiwan; 5 Department of Pathology, Changhua Christian Hospital, Changhua, Taiwan; 6 Graduate Institute of Cancer Biology and Drug Discovery, Taipei Medical University, Taipei, Taiwan

## Abstract

**Background:**

A thymine/cytosine point mutation in the MSP I restriction site of cytochrome P450 1A1 (*CYP1A1*) has been linked to susceptibility to smoking-related cancers and is reported to result in increased enzyme activity. Therefore, we sought to determine whether allelic variation of *CYP1A1* is associated with protein expression and protein activity in pterygium.

**Methods:**

We collected 150 pterygium samples and 50 normal conjunctiva samples, which served as controls. DNA samples were extracted from blood cells and then subjected to real-time ploymerase chain reaction (PCR) to determine *CYP1A1* genotype. CYP1A1 protein expression was determined by immunohistochemical staining with a monoclonal antibody for CYP1A1. Pterygium epithelial cells (PECs), cultured in a serum-free culture medium, real-time PCR, western blot and enzyme-linked immunosorbent assay (ELISA) were used to understand the effect of *CYP1A1* allelic variation in protein expression and activity.

**Results:**

Forty-eight (33.3%) pterygium specimens tested positive for CYP1A1 protein expression. CYP1A1 protein expression was significantly greater in the pterygium group than in the control group (p<0.0001). In addition, CYP1A1 protein expression was associated with allelic variation. CYP1A1 protein expression was significantly greater in the m2/m2 group than in the m1/m1and m1/m2 groups (p=0.006). In the cell model, CYP1A1 protein expression and b[a]P 7,8-diol 9,10-epoxide (BPDE)-like DNA adducts increased in *CYP1A1* m2/m2 (genotype T/T) PEC cells as compared with m1/m2 (genotype C/T) and m1/m1 (genotype C/C) cells.

**Conclusions:**

CYP1A1 expression in pterygium correlates with allelic variation and can be used as an independent risk marker.

## Introduction

Pterygium used to be known as a degenerative disease. The p53 protein was found to be abnormally expressed in the epithelium of pterygia; consequently, pterygium is now considered to be a ultra violet (UV) exposure–related, uncontrolled cell proliferation similar to a tumor [1-7].

Benzo[a]pyrene (BaP) is oxidized by a series of well characterized enzymes, such as cytochrome p450 1A1, 2C9, and 3A4 [8,9]. A thymine/cytosine point mutation in the MSP I restriction site of cytochrome P450 1A1 (*CYP1A1*) has been reported to result in increased enzyme activity [10]. The *CYP1A1* MspI allelic variant has been linked to susceptibility to smoking-related cancers, such as oral, colon, breast, and lung cancers [11-13]. We previously reported that benzo[a]pyrene (B[a]P) 7,8-diol 9,10-epoxide (BPDE)-like DNA adducts detected in pterygium samples were associated with allelic variants of *CYP1A1* [14]. The risk of BPDE-like DNA adduct formation in patients with *CYP1A1* m2/m2 and m1/m2 was 9.675 fold greater than in patients with m1/m1 types [14]. We also found that the risk of pterygium formation was 2.339 fold greater with the *CYP1A1* m2/m2 genotype as compared with the C/T and T/T genotypes [15]. Therefore, allelic variants of *CYP1A1* may contribute to BPDE-DNA adduct formation and pterygium progression.

The association of CYP1A1 protein and genetic polymorphism have been reported in several types of cancer, but no any report was discussed about this in pterygium. Therefore, in this study, we analyze the association of *CYP1A1* allelic variants with protein expression using real-time PCR and immunohistochemistry methods with 150 pterygium specimens and 50 normal conjunctiva samples. Cell models were used to confirm the association of *CYP1A1* allelic variants with protein expression and activity.

## Methods

### Study subjects

Pterygium samples were harvested from 150 surgical patients in whom the apex of the pterygium had invaded the cornea by more than 1 mm. The controls included normal conjunctiva samples collected from the superior conjunctiva of 20 patients and the medial conjunctiva of 30 patients without pterygium or pinguecula; control subjects were undergoing cataract or vitreoretinal surgery. Normal conjunctiva samples were collected from the bulbar conjunctiva. There were 87 males and 63 females in the pterygium group (age range=55–84 years, mean=66.3 years), and there were 25 males and 25 females in the control group (age range=55–78 years, mean=65.7 years). There were no significant difference in age and gender between control and pterygium groups. All pterygium specimens came from primary pterygia. All specimens were fixed in formalin and embedded in paraffin. Blood samples of primary pterygia and control groups were also collected in this study to be used in genotype analysis. All subjects provided written informed consent, and the study was approved by the institutional review board.

### Polymorphisms of *CYP1A1*

DNA was extracted from the blood cells of the pterygium and control groups for genetic polymorphism analysis [14,15]. DNA lysis buffer was applied to lyse the epithelial cells on a slide, and then the DNA solution was transferred to an Eppendorf tube for traditional proteinase K digestion and phenol-chloroform extraction. Finally, the DNA was precipitated by ethanol; linear polyacrylamide was added to increase the length of the DNA fragments [16]. Genotyping of the samples were was performed by real time- ploymerase chain reaction (PCR) using the primer set 5′-TAG GAG TCT TGT CTC AGC CT-3′ and 5′-CAG TGA AGA GGT GTA GCC GCT-3′ [17]. The *CYP1A1* gene was amplified by PCR in a total reaction volume of 25 μl, containing 2.5 μl 10× PCR buffer (Mg^2+^ Plus), 0.5 μl 10 mM dNTP Mixture, 0.5U Taq DNA polymerase (TaKaRa Co. Ltd., Dalian, China), 100 ng DNA template, each of 1 μl (10 μM) primers, and an appropriate amount of purified water. The PCR proceeded under the following conditions: pre-denaturing at 95 °C for 5 min, 30 cycles at 94 °C for 1 min, 60 °C for 1 min and 72 °C for 1 min, and at 72 °C for 10 min. PCR products were digested with excess MspI restriction enzyme (TaKaRa Biotech. Co. Ltd.) at 37 °C overnight in a water bath, and then electrophoresized in 1.5% agarose gels and visualized by ethidium bromide staining on UV transilluminator (M-20, UVP; LLC, Upland, CA). The MspI restriction site polymorphism resulted in 3 genotypes: a predominantly homozygous m1 allele without the MspI site (genotype m1/m1; C/C; 899 bp product), the heterozygote (genotype m1/m2; C/T; 899 bp, 693 bp, 206 bp products), and a rare homozygous m2 allele with the MspI site (genotype m2/m2; T/T; 693 bp, 206 bp products).

### Immunohistochemistry

Formalin-fixed and paraffin-embedded specimens were sectioned at a thickness of 3 μm. All sections were deparaffinized in xylene, sequentially rehydrated through serial dilutions of alcohol, and washed in phosphate-buffered saline. Sections used for CYP1A1 detection were heated in a microwave oven twice for 5 min in a citrate buffer (pH 6.0). The primary antibodies were mouse anti- CYP1A1 monoclonal antibodies (at a dilution of 1:200; Chemicon International, Temecula, CA). The primary antibody was mouse anti-CYP1A1 monoclonal antibody (at a dilution of 1:200; Chemicon International, Temecula, CA) and the incubation time was 60 min at room temperature followed by a conventional streptavidin peroxidase method (LSAB Kit K675; DAKO, Copenhagen, Denmark). Signals were developed with 3, 3'-diaminobenzidine for 5 min and counter-stained with hematoxylin [18,19]. Negative controls that did not include the primary antibodies were also prepared. The results were independently evaluated by 3 observers and were scored for the percentage of cells with positive expression. Scores were indicated as follows: 0, no positive staining; +, 1%–10%; ++, 11%–50%; and +++, more than 50% of cells with positive staining. In this study, scores of +, ++, and +++ were considered representative of positive immunostaining, and a score of 0 was classified as negative immunostaining.

### Pterygium cell lines

Pterygium cell lines (PECs) used in this study were established previouly [20]. Fresh pterygial specimens were cut into small pieces (1–2 mm in diameter) under a stereomicroscope, washed in DMEM (HyClone, Logan, UT) solution, and placed in a culture dish. Serum free DMEM culture medium (HyClone) was added to cover the explants. The culture dish was put in a CO_2_-regulated incubator with a 5% CO_2_ atmosphere overnight. The culture medium was replaced three times a week after the appearance of an outgrowth of cells from the explants. To confirm that whether the established PECs cells were epithelial cells, the cells was further confirmed using p63 and pan cytokeratin antibodies [20] (Santa Cruz Biotechnology, Santa Cruz, CA).

### Western blot

The primary PECc cells (pterygium epithelial cells) used in this study were culture within 10 passage. The genotype of these cells was first identified by polymerase chain reaction-restriction fragment length polymorphism (PCR-RFLP). Total proteins were extracted from PECs cells with a lysis buffer (100 mM Tris, pH 8.0, 1% SDS), and recovered protein concentrations were determined using the Bio-Rad protein assay kit (Bio-Rad Laboratories, Inc., Hercules, CA) followed by separation with sodium sodecyl sulfate polyacrylamide gel electrophoresis (SDS-PAGE; 12.5% gel, 1.5 mm thick). After the electrophoretic transfer to a Hybond-polyvinylidene difluoride (PVDF; Millipore, Billerica, MA) membrane, nonspecific binding sites were blocked with 5% nonfat milk in TBS-Tween-20. The detection of CYP1A1, p63, pan cytokeratin and β-actin was conducted by incubating the membrane with anti-CYP1A1 (Chemicon International), p63 and pan cytokeratin (Santa Cruz Biotechnology, Santa Cruz, CA) and β-actin antibodies (Sigma -Aldrich, Lyon, France) for 60 min at room temperature, followed by subsequent incubation with a peroxidase-conjugated secondary antibody (1:5000 dilution; Sigma). Extensive washings with TBS-Tween-20 were performed between incubations to remove non-specific binding. The protein bands were visualized using enhanced chemiluminescence (NEN Life Science Products Inc., Boston MA). Densitometer analysis of films was performed using a computerized image analysis (AlphaImager HP; Alpha Innotech, San Leandro, CA) program. Protein expression levels were established by calculating the target molecule/β-actin ratio (all cases scored for band intensity compared with internal control). Expression intensity of 5% or less of control levels was interpreted as negative.

### Competitive color ELISA

The ELISA method used in this study for detecting polycyclic aromatic hydrocarbon-DNA adducts was a modification of the competitive color ELISA described previously [19,21,22]. Microtiter plates were coated with 3 ng of highly modified BPDE-DNA in PBS, by drying at 37 °C overnight. Coated plates were washed initially and between each step with PBS containing 0.1% Tween-20. Plates were then blocked for nonspecific binding by incubation with PBS-Tween containing 1% fetal calf serum at 37 °C for 60 min to block nonspecific binding. BPDE-DNA standards and DNA samples (25 µg in 50 µl/well, in duplicate) were mixed with an equal volume (50 µl) of polyclonal antibody HL1 (1:5×10^5^ dilution) [19] and added to the plate. Microtiter plates were coated with highly modified BPDE-DNAPlates were then blocked for nonspecific binding by incubation with PBS-Tween containing 1% fetal calf serum. BPDE-DNA standards and DNA samples were mixed with an equal volume of polyclonal antibody HL-1 (1:5×10^5^ dilution) [19] and added to the plate. After incubation at 37 °C for 60 min, goat anti-rabbit IgG alkaline phosphatase conjugate (100 µl, 1:1000 dilution) was added, and the plates were incubated at 37 °C for 90 min. The plates were washed, and the substrate, p-nitrophenyl phosphate (100 µl of 1 mg/ml in 1M diethanolamine, pH 8.6), was added and the color at 405 nm was measured by a microtiter plate reader (Model 550; BIO-RAD, Hercules, CA). The percentage of inhibition values for the DNA samples were calculated by comparison with the non-modified calf thymus DNA control. A standard curve of BPDE-DNA standard (from 0.33 to 328.6 fmol/well) was used to determine the DNA-adduct levels in the samples. Samples showing less than 20% inhibition were considered not detectable.

### Statistical analysis

Statistical analysis was performed using the SPSS statistical software program (SPSS, Inc., Chicago, IL). The Fisher exact test and χ^2^ test were used. Adjusted odd ratios (ORs) and 95% confidence intervals (CIs) for various factors in the pterygium subjects were evaluated using a multiple logistic regression model. A p<0.05 was considered to be statistically significant.

## Results

### CYP1A1 protein expression in pterygia and conjunctivas

**Figure 1:**
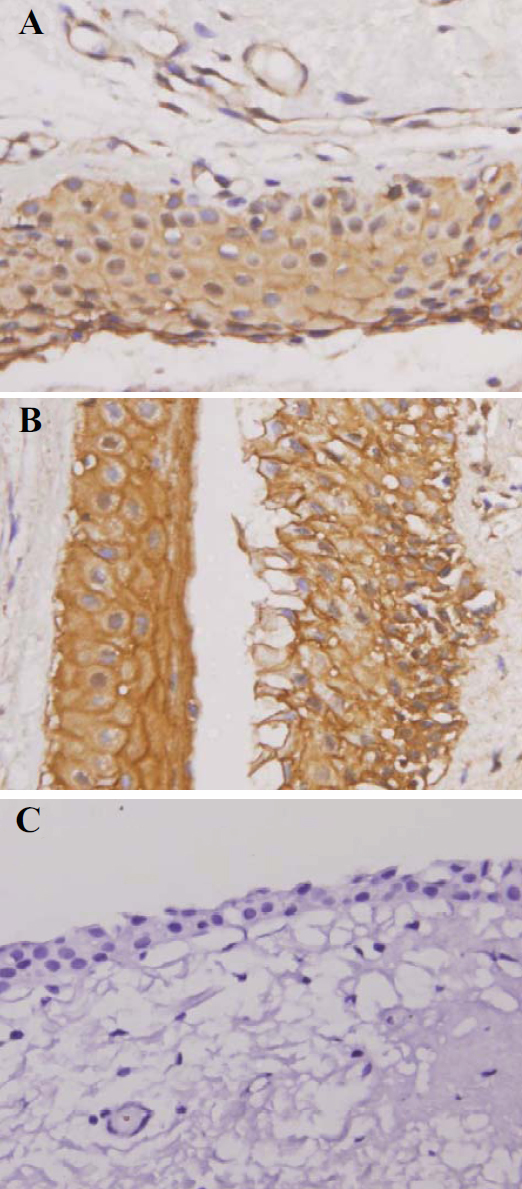
Representative positive and negative immunostainings for CYP1A1 protein in paraffin sections of pterygium. Representative positive CYP1A1 immunostainings are shown in (**A**) and (**B**; 400×), negative immunostainings are shown in (**C**; 400×), representative.

Immunohistochemistry was used to analyze CYP1A1 protein expression in 150 pterygium and 50 conjunctiva samples (Figure 1). In the pterygium group, 48 (33%) samples were positive for CYP1A1 expression. CYP1A1 staining was distributed in the cytoplasm of epithelial and subepithelial connective tissues (Table 1). Only 4% (2 of 50) of the normal conjunctiva samples were positive for CYP1A1 protein expression. CYP1A1 expression was greater in the pterygium group than in the control group (p<0.0001).

**Table 1:** Immunohistochemical analysis of CYP1A1 protein expression in pterygium (n=150) and control^a^ (n=50) tissues, p<0.0001.

**CYP1A1 result**	**Pterygia, n (%)**	**Controls, n (%)**
Negative	102 (67.0)	48 (96.0)
Positive	48 (33.0)	2 (4.0)

^a^From superior conjunctiva of 20 patients and medial conjunctiva of 30 patients without pterygium or pinguecula.

### *CYP1A1* allelic variants in pterygium and control groups

To verify the association between risk and genetic change in the metabolic genes in pterygium development, the allelic variants of the *CYP1A1* gene in the pterygium and control groups were analyzed. Table 2 presents *CYP1A1* genotypes of the pterygium and control groups. In the pterygium group, 50 (33.3%) were homozygous for the m1/m1 genotype, 22 (14.7%) were homozygous for the m2/m2 genotype, and 78 (52.0%) were heterozygous for the m1/m2 genotype. In the control group, 26 (52.0%) were homozygous for the m1/m1 genotype, 8 (16.0%) were homozygous for the m2/m2 genotype, and 16 (32.0%) were heterozygous for the m1/m2 genotype. There was a statistically significant difference between the *CYP1A1* genotypes of the pterygium and control groups (p=0.035).

**Table 2:** *CYP1A* genotype^a^ distribution among pterygium (n=150) and control^b^ (n=50) subjects, p=0.035

**Genotype**	**Pterygium, n (%)**	**Control, n (%)**
m1/m1	50 (33.3)	26 (52.0)
m1/m2	78 (52.0)	16 (32.0)
m2/m2	22 (14.7)	8 (16.0)

^a^*CYP1A1* allelic variants analyzed in this study are based on prior pterygium samples [14,15]. ^b^From superior conjunctiva of 20 patients and medial conjunctiva of 30 patients without pterygium or pinguecula.

### The association of *CYP1A1* allelic variants with protein expression

As shown in Table 3, CYP1A1 protein expression in the m2/m2 group was significantly greater than in the m1/m1 and m1/m2 groups (p=0.027). No significant association was found in the control group. Therefore, we suggest that CYP1A1 protein expression in pterygium tissues is associated with allelic variants.

**Table 3:** Association between allelic variation^a^ and CYP1A1 protein expression in pterygium (n=150) and control^b^ (n=50) subjects

** **	**CYP1A1 protein expression, n (%)**		** **
**Genotype**	**Negative**	**Positive**	**p**
Pterygium	** **	** **	0.027
m1/m1	40 (39.2)	10 (20.8)	** **
m1/m2 and m2/m2	62 (60.8)	38 (79.2)	** **
Control	** **	** **	1.000
m1/m1	25 (52.1)	1 (50.0)	** **
m1/m2 and m2/m2	23 (47.9)	1 (50.0)	** **

^a^*CYP1A1* allelic variants analyzed in this study are based on prior pterygium samples [14,15]. ^b^From superior conjunctiva of 20 patients and medial conjunctiva of 30 patients without pterygium or pinguecula. The CIP 1A1 protein in pterygium and conjunctiva controls was detected by immunohistochemistry.

### The risk of CYP1A1 protein expression in pterygium progression

We compared CYP1A1 protein expression in the pterygium and control groups. After adjustment for age and sex, multiple logistic regression analysis showed that the *CYP1A1* genotype is related to pterygium. The prevalence of pterygium in subjects with CYP1A1 protein expression appeared to be greater than the prevalence of pterygium in those without CYP1A1 protein expression (OR 11.49, 95% CI 2.60–5.0, p=0.001, Table 4). There was no statistically significant difference in sex (p=0.098), but older individuals showed a significantly increased prevalence of pterygium relative to younger individuals (OR 2.56, 95% CI 1.28–5.13, p=0.008, Table 4).

**Table 4:** Multiple logistic regression analysis of *CYP1A1* genotype, age, sex, and risk of pterygium.

**Variable**	**Groups unfavorable/favorable**	**OR (95% CI)**	**p**
*CYP1A1*	wild type/allelic variant	11.49 (2.60–5.00)	0.001
Sex	female/male	1.81 (0.90–3.65)	0.097
Age	<65/≥65	2.56 (1.28–5.13)	0.008

### The association of *CYP1A1* allelic variants and protein expression in PEC cells

**Figure 2:**
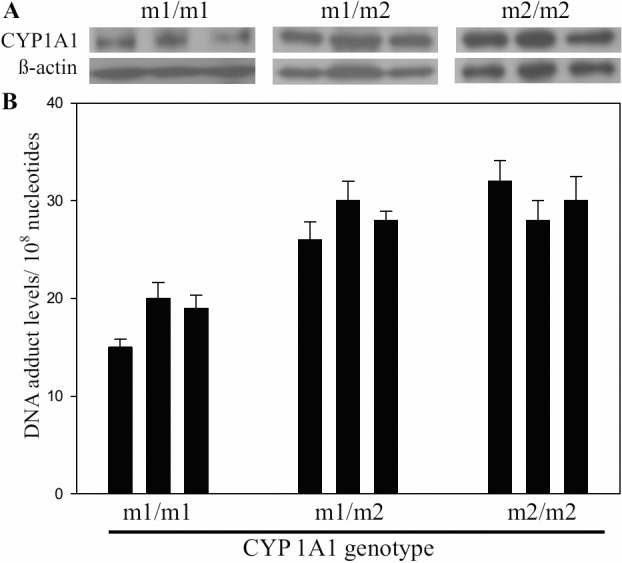
The association of *CYP1A1* allelic variants, protein expression and Bap-like DNA adduct levels in PEC cells. **A**: The representative CYP1A1 protein expression in PEC cells with different *CYP1A1* genotypes. **B**: BPDE-like DNA adduct levels in PEC cells detected by ELISA. Data showed that PEC cells with m1/m2 and m2/m2 genotypes were significantly higher than in the cells with m1/m1 genotype.

To verify whether the CYP1A1 protein activity in pterygium is associated with genetic allelic variation, we established primary cultured epithelial cell lines (PEC cells) with different *CYP1A1* genotypes from the pterygium patients. The cell type was confirmed by p63 and pan cytokeratin staining to demonstrate that they were epithelial cells. The *CYP 1A1* genotype and protein expression of these cell lines were detected by real-time PCR and western blot. The results showed that the protein expression in the m1/m1 genotype was significantly lower than in the m1/m2 and m2/m2 genotypes. But no differences were found between the m1/m2 and m2/m2 genotypes (Figure 2A). To further confirm the CYP1A1 protein activity could be affected by genotype, PEC cells with different genotypes were treated with B[a]p and the BPDE-like DNA adducts were evaluated by ELISA. Our data demonstrated that the BPDE-like DNA adduct levels in PEC cells with m1/m2 and m2/m2 genotypes were significantly higher than in the cells with m1/m1 genotype (Figure 2B). Therefore, we suggest that the activity of CYP1A1 protein was correlated with the genetic allelic variant.

## Discussion

Our previous study showed that BPDE-like DNA adduct levels correlate with *CYP1A1* allelic variation in pterygium [14]. We also found that the risk of pterygium is 2.339 fold greater for individuals with the m2/m2 (C/C) genotype compared with individuals with the C/T (m1/m2)and T/T (m1/m1) genotypes [15]. Therefore, we hypothesize that after exposure to environmental PAHs, the *CYP1A1* allelic variation may result in high levels of BPDE-like DNA adduct formation, contributing to the risk of pterygium.

The *CYP1A1* MSPI allele may be a risk factor for pterygium. It has been reported that a thymine/cytosine point mutation in the MSP I restriction site of *CYP1A1* results in increased enzyme activity [10]. Using pterygium tissue to test for CYP1A1 protein activity is difficult. Uppstad et al. [23], using ethoxyresorufin-O-deethylase (EROD) analysis, demonstrated that CYP1A1 protein activity correlates with gene expression levels in lung cancer cell lines. In addition, several studies also found that CYP1A1 protein activity in human tissues correlates with gene expression levels [24,25]. In this study, we tested the association between allelic variants of *CYP1A1* and protein expression. To our knowledge, this is the first study to analyze the association between allelic variants and CYP1A1 protein expression in pterygium. We found that CYP1A1 protein expression of the m2/m2 genotype was significantly greater than that of the m1/m1 genotype, but not greater than that of the m1/m2 genotype (Table 3). In addition, CYP1A1 protein expression in the pterygium group was significantly greater than in the control group (Table 1). CYP1A1 expression and DNA adduct levels were found to have a statistically significant association [25,26]. In our study, a similar staining pattern was also found in cell model experiments, and the CYP1A1 protein activity correlated with the genetic variant (Figure 2). Therefore, we suggest that allelic variation of *CYP1A1* contributes to protein expression in pterygium and results in high levels of BPDE-like DNA adduct formation.

In summary, we found that CYP1A1 protein expression in pterygium tissue was significantly greater than in nonpterygium control tissues, and CYP1A1 protein expression is associated with allelic variation. In addition, CYP1A1 protein expression can be used as an independent risk factor. Therefore, we suggest that CYP1A1 protein expression in pterygium contributes to BPDE-like DNA adduct formation.
